# Hemiparesis Caused by Cervical Spontaneous Spinal Epidural Hematoma: A Report of 3 Cases

**DOI:** 10.4061/2011/516382

**Published:** 2011-07-28

**Authors:** Kinya Nakanishi, Naoki Nakano, Takuya Uchiyama, Amami Kato

**Affiliations:** Department of Neurosurgery, Kinki University School of Medicine, 377-2 Onohigashi, Osakasayama-shi, Osaka 589-8511, Japan

## Abstract

We report three cases of spontaneous spinal epidural hematoma (SSEH) with hemiparesis. The first patient was a 73-year-old woman who presented with left hemiparesis, neck pain, and left shoulder pain. A cervical MRI scan revealed a left posterolateral epidural hematoma at the C3–C6 level. The condition of the patient improved after laminectomy and evacuation of the epidural hematoma. The second patient was a 62-year-old man who presented with right hemiparesis and neck pain. A cervical MRI scan revealed a right posterolateral dominant epidural hematoma at the C6-T1 level. The condition of the patient improved after laminectomy and evacuation of the epidural hematoma. The third patient was a 60-year-old woman who presented with left hemiparesis and neck pain. A cervical MRI scan revealed a left posterolateral epidural hematoma at the C2–C4 level. The condition of the patient improved with conservative treatment. The classical clinical presentation of SSEH is acute onset of severe irradiating back pain followed by progression to paralysis, whereas SSEH with hemiparesis is less common. Our cases suggest that acute cervical spinal epidural hematoma should be considered as a differential diagnosis in patients presenting with clinical symptoms of sudden neck pain and radicular pain with progression to hemiparesis.

## 1. Introduction

Spontaneous spinal epidural hematoma (SSEH) is uncommon, but the number of cases has increased with clarification of the clinical presentation of the condition using radiographic imaging. Here, we report three cases of SSEH with hemiparesis, which is uncommon compared to the classical presentation of SSEH as acute onset of severe irradiating back pain followed by paralysis.

## 2. Case Presentation

### 2.1. Case 1

**Figure 1:**
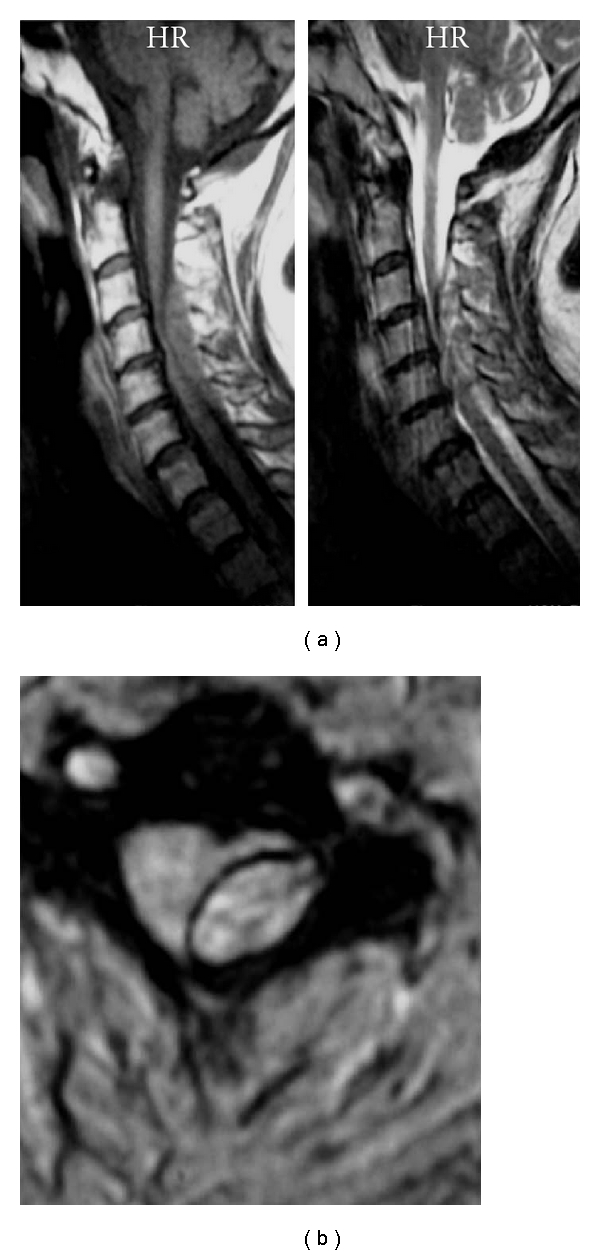
Preoperative sagittal (a) and axial (b) MR images showing a left posterolateral epidural hematoma at the C3-C6 level with spinal cord compression.

The patient was a 73-year-old woman who experienced acute onset of severe pain in the back of her neck with radiation into her left shoulder. Over the next day, she developed left hemiparesis and was admitted to our hospital. An examination showed left hemiparesis (left upper and lower extremities; manual muscle testing (MMT) 1/5) with numbness in the left upper and lower extremities, without facial palsy, dysarthria, and aphasia. Deep-tendon reflexes were hypoactive on the left side with a left Babinski reflex. A head CT scan was normal, but a cervical MRI scan revealed a left posterolateral epidural hematoma at the C3–C6 level (Figure 1). 24 hours after onset, right hemilaminectomy from C3 to C5 and evacuation of the epidural hematoma were performed. One day after surgery, the patient improved to MMT 3/5, after 2 weeks improved to MMT 4/5 in the left upper and lower extremities, and at the 2-year followup, the patient continued left hemiparesis (MMT 4/5).

### 2.2. Case 2

**Figure 2:**
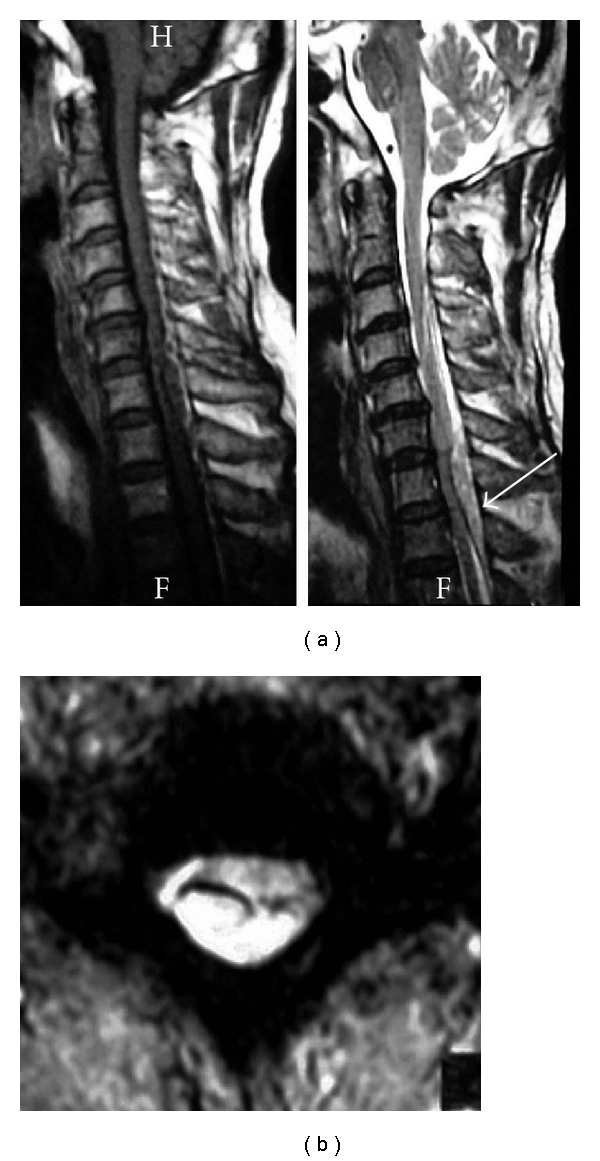
Preoperative sagittal (a) and axial (b) MR images showing a right posterolateral dominant epidural hematoma at the C6-T1 level with spinal cord compression arrow.

The patient was a 62-year-old man who experienced sudden pain of the posterior cervical region and numbness of the right lower extremity when he bent backward to administer eye drops. Subsequently, paralysis developed in the right upper and lower extremities, and he visited the emergency room of our hospital with suspicion of a cerebral stroke. An examination showed right hemiparesis (right upper and lower extremities; MMT 2/5) with numbness in the right upper and lower extremities and bladder and rectal disturbance, without facial palsy, dysarthria, and aphasia. Deep-tendon reflexes were hyperactive in the lower extremities with a right Babinski reflex. Head CT and MRI were normal, but cervical MRI showed a right dominant posterolateral spinal epidural hematoma at the C6-T1 level (Figure 2). 6 hours after onset, hemilaminectomy from C6 to T1 and evacuation of the epidural hematoma were performed. One day after surgery, the patient improved to MMT 5/5 in the right upper and lower extremities, at followup of 2 years he had no right hemiparesis. 

### 2.3. Case 3

**Figure 3:**
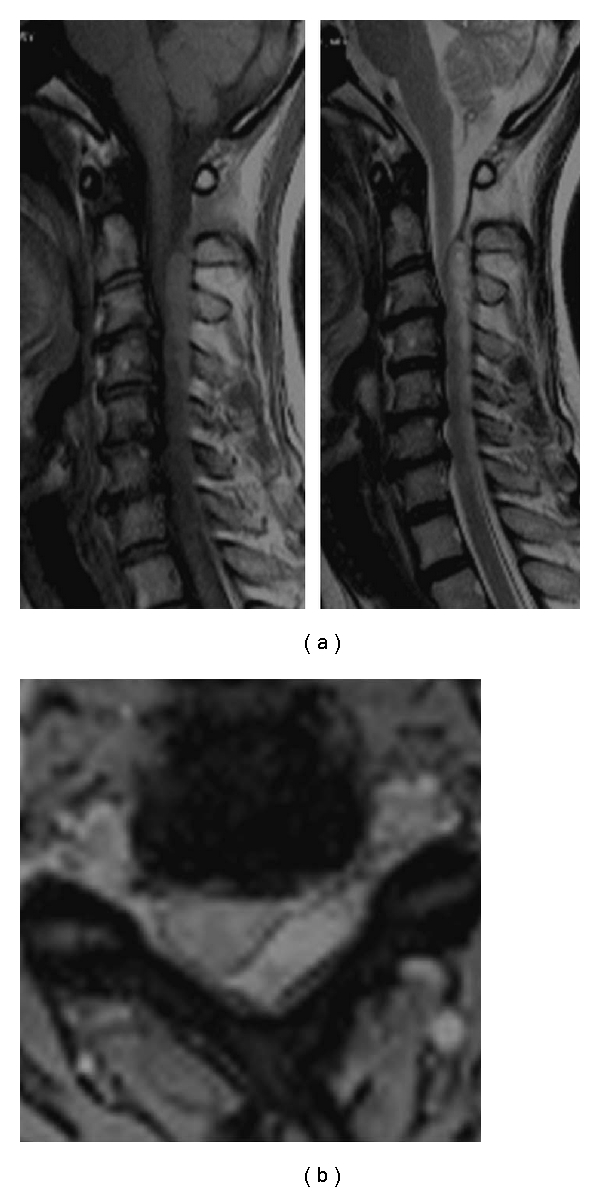
Initial sagittal (a) and axial (b) MR images showing a left posterolateral epidural hematoma at the C2-C4 level with spinal cord compression.

A 60-year-old woman developed sudden pain of the posterior cervical region during a conversation. The pain aggravated gradually, and she developed left hemiparesis approximately 20 minutes after the onset of pain. Head CT and MRI were normal, but cervical MRI showed a spinal epidural hematoma limited to the left spinal dorsal region at the C2–C4 level (Figure 3). An examination on the first visit to our hospital did not indicate muscle weakness or sensory disturbance. Deep-tendon reflexes of the four extremities were increased mildly, and the Babinski reflex was absent on both sides. In the clinical course, left hemiparesis improved rapidly and conservative treatment was selected. Cervical MRI performed 2 weeks after onset showed complete disappearance of the epidural hematoma.

## 3. Discussion

Spinal epidural hematoma was first described by Jackson [1] in 1869. The yearly incidence is now thought to be approximately 0.1 per 100,000 people [2], and the condition is no longer considered to be rare due to increased diagnosis by MRI. As possible etiology factors, minor trauma, sneezing, whooping cough, voiding, vomiting, lifting, pregnancy, hypertension, atherosclerosis, anticoagulants, and bleeding diathesis have been mentioned [3, 4]. Analysis of a large series of SSEH that were reported in the international medical literature suggested a correlation between SSEH and coexistence of arterial hypertension [3]. These factors may cause secondary spinal epidural hematoma with a clear cause (60%) or spontaneous spinal epidural hematoma (SSEH) of unclear cause (40%) [3, 5, 6]. SSEH tends to be more common in middle-aged or older patients, in males compared to females [3]. The majority of SSEH are situated in the C5-Th2 area [3].

The mechanism of development of SSEH is unclear. It has been suggested that venous pressure may increase in line with an increase in abdominal and intrathoracic pressure, since the spinal vein is of the primitive type with no venous valve, and that this may easily cause hemorrhage [7, 8]. Alternatively, SSEH may develop due to a collapse of the free epidural artery following hemorrhage at a level that causes spinal cord compression, with acute onset and progress of symptoms [5]. Many reports have also suggested that SSEH may be triggered by actions that increase venous pressure, such as cough, sneezing, and holding of heavy baggage [9], and venous hemorrhage has also been proposed as a cause. Gradual progress from development of pain to hemiparesis caused by venous hemorrhage occurred in the three cases reported here. The majority of SSEH are situated posterior or posterolaterally in the spinal canal [4]. The morphological pattern of anterior internal vertebral venous plexus (IVVP) in the human fetus and in aged human cadavers is identical. In contrast to the situation in the aged human, the lower thoracic and lumbar posterior IVVP in the fetus is very small and lacks prominent transverse venous bridges. The morphological differences seem to give a clue to the origin of the SSEH; in SSEH, an age-related segmental distribution of the hematomas has been observed, which might be related to the morphological changes that occur within the posterior IVVP during the process of aging [4, 10]. 

Development of SSEH is characterized by symptoms of sudden cervical or back pain followed rapidly by motor paralysis or anesthesia [7, 11]. SSEH with hemiparesis is less common but has been reported previously. In addition to back pain, Groen and Goffin [7] reported symptoms of hemiparalysis in 193 cases (58%), paralysis/anesthesia in 123 cases (37%), nerve root symptoms in 15 cases (4%), and unclear symptoms in 2 cases (1%) in a review of 333 cases of spinal epidural hematoma. Hemiparesis has also been reported in 2 of 10 patients with SSEH described by Kimiwada et al. [9], in 6 of 35 patients in Liao et al. [12], and in 2 of 4 patients in Lonjon et al. [13]. Collectively, these reports show that hemiparesis is not uncommon in SSEH patients. Our cases suggest that acute cervical spinal epidural hematoma should be considered as a differential diagnosis in patients presenting with clinical symptoms of sudden neck pain and radicular pain with progression to hemiparesis.
